# Haploidentical donor stem cell transplantation had a lower incidence of bronchiolitis obliterans syndrome compared with HLA-matched sibling donor transplantation in patients with hematologic malignancies: Benefit from ATG?

**DOI:** 10.3389/fimmu.2022.1036403

**Published:** 2022-10-27

**Authors:** Guangyang Weng, Zhiping Fan, Huiwen Xue, Fen Huang, Na Xu, Hua Jin, Sijian Yu, Zhixin Ye, Jingchao Fan, Li Xuan, Qifa Liu

**Affiliations:** ^1^ Department of Hematology, Nanfang Hospital, Southern Medical University, Guangzhou, China; ^2^ Department of Hematology, First Affiliated Hospital of Shenzhen University, The Second People’s Hospital of Shenzhen, Shenzhen, China

**Keywords:** antithymocyte globulin, bronchiolitis obliterans syndrome, haploidentical donor stem cell transplantation, graft, GVHD

## Abstract

**Background:**

Haploidentical donor stem cell transplantation (HID-SCT) based on antithymocyte globulin (ATG) for graft-versus-host disease (GVHD) prophylaxis had achieved a similar incidence of chronic graft-versus-host disease (cGVHD) with human leukocyte antigen (HLA)-matched sibling donor stem cell transplantation (MSD-SCT). However, bronchiolitis obliterans syndrome (BOS), which serves as pulmonary cGVHD, was rarely compared between HID and MSD transplantation.

**Methods:**

One thousand four hundred five patients with hematologic malignancies who underwent allogeneic SCT were enrolled in this retrospective study. Based on donor type, we divided the patients into three groups: HID, MSD, and match unrelated donor (MUD) groups. The cumulative incidences and risk factors of BOS were analyzed.

**Results:**

The 5-year cumulative incidence of BOS was 7.2% in the whole population. HID transplantation had a lower 5-year cumulative incidence of BOS than MSD transplantation (4.1% vs. 10.0%, p < 0.001) and a similar incidence with MUD transplantation (4.1% vs. 6.2%, p = 0.224). The 5-year cumulative incidence of BOS was lower in the ATG group than that in the non-ATG group in both the whole and MSD populations (4.6% vs. 11.2%, p < 0.001, and 4.1% vs. 11.2%, p = 0.042, respectively). The 5-year incidence of BOS in mixed grafts [peripheral blood stem cell (PBSC) plus bone marrow] group was also lower than that in the PBSC group (4.2% vs. 9.1, p = 0.001). Multivariate analysis showed that HID, ATG, and mixed grafts were protective factors for BOS [odds ratio (OR) 0.3, 95% CI 0.2–0.6, p < 0.001; OR 0.3, 95% CI 0.2–0.7, p = 0.001; OR 0.3, 95% CI 0.1–0.8, p = 0.013], and acute graft-versus-host disease (aGVHD) and cGVHD were independent risk factors for BOS (OR 2.1, 95% 1.1–4.3, p = 0.035; OR 10.1, 95% CI 4.0–25.0, p < 0.001).

**Conclusions:**

HID transplantation had a lower incidence of BOS than MSD transplantation, which might be associated with ATG and mixed grafts.

## Introduction

Haploidentical donor stem cell transplantation (HID-SCT) is widely used to treat hematologic malignancies and achieves similar outcomes compared with human leukocyte antigen (HLA)-matched sibling donor stem cell transplantation (MSD-SCT) (1–4). Historically, alloreactivity generated by HLA disparity led to severe graft-versus-host disease (GVHD) and limited the application of HID-SCT (5–7). Luckily, application of antithymocyte globulin (ATG) or posttransplantation cyclophosphamide (PT-Cy) for GVHD prophylaxis has overcome this barrier. Now, HID-SCT becomes the main alternative option for patients lacking an MSD or HLA-matched unrelated donor stem cell transplantation (MUD-SCT) (8–10).

An increasing number of studies suggest that ATG may reduce the incidence of chronic graft-versus-host disease (cGVHD) regardless of HID, MUD, or MSD transplantation (11–15). Moreover, the incidences of cGVHD were comparable between HID transplantation based on ATG and MSD transplantation (15–17). There is even some evidence suggesting that HID transplantation based on ATG has a lower incidence of cGVHD than MSD transplantation (18, 19). Bronchiolitis obliterans syndrome (BOS) is a fatal complication and considered a manifestation of pulmonary cGVHD, with a 2%–14% incidence and only 13%–56% in 5-year overall survival (OS) (20). Now, it is unclear whether the incidence of BOS is lower in HID transplantation based on ATG than that in MSD transplantation. Thus, we retrospectively analyzed the incidence and risk factors of BOS in a cohort of 1,405 patients with hematologic malignancies who underwent allogeneic stem cell transplantation (allo-SCT). Our study showed that HID transplantation had a lower incidence of BOS than MSD transplantation, which might be associated with ATG and mixed grafts [peripheral blood stem cell (PBSC) plus bone marrow (BM)].

## Patients and methods

### Patients

This was a single-center retrospective study. Patients with hematologic malignancies undergoing allo-SCT and myeloablative conditioning and surviving more than 100 days posttransplantation were enrolled at the Nanfang Hospital between 1 June 2009 and 30 October 2019. The Ethics Committee of Nanfang Hospital approved this study.

### Transplantation

MSD was preferred, followed by an HLA-matched MUD. If both of these donor types were unavailable, patients would receive a transplantation from an HID (3). Two kinds of myeloablative conditioning regimens were administered, including busulfan (BU)- and Total Body Irradiation (TBI)-based regimens. In general, BU-based regimens were used in patients with myelogenous malignancies in complete remission and TBI-based regimens were used in patients with lymphocytic malignancy or patients in non-complete remission. The detailed regimens were shown as previously described (15, 21, 22). The majority of HID-SCT recipients transplanted with mixed grafts, whereas most MSD-SCT and all MUD-SCT recipients received PBSC grafts.

### Graft-versus-host disease prophylaxis

As indicated in our previous studies (15, 22, 23), ciclosporin A (CsA) and methotrexate (MTX) were administered to patients undergoing MSD transplantation for GVHD prophylaxis, and mycophenolate mofetil (MMF) was added to GVHD prophylaxis of MSD transplantation from June 2013. CsA + MTX + ATG (Thymoglobulin; Genzyme, Cambridge, MA, USA) (total ATG dose, 7.5 mg/kg on days -3 to -1) were administered to patients undergoing MUD transplantation, and CsA + MTX + ATG (total ATG dose, 7.5 or 10 mg/kg on days -3 to 0) + MMF were administered to patients undergoing HID transplantation for GVHD prophylaxis. ATG (total ATG dose, 4.5 mg/kg on days -3 to -1) was applied to a minority of MSD-SCT recipients (12).

### Diagnosis and treatment of bronchiolitis obliterans syndrome

Pulmonary function testing (PFT) was routinely performed in all patients before transplantation. Posttransplant PFT was conducted in patients with unexplained respiratory symptoms at ≥100 days posttransplantation, such as significant dyspnea on exertion, decreased exercise tolerance, and a persistent nonproductive cough. BOS was clinically diagnosed according to modified National Institutes of Health (NIH) criteria (24): 1) Forced Expiratory Volume in 1 second (FEV1)/vital capacity <0.7; 2) FEV1 <75% predicted along with 10% decline from the pretransplantation baseline; 3) absence of active respiratory infections; 4) one of the following manifestations: evidence of air trapping by expiratory CT or small airway thickening or bronchiectasis by high-resolution chest CT; residual volume (RV) >120% of predicted or RV/total lung capacity (TLC) elevated outside the 90% confidence interval (RV/TLC). The combination of corticosteroids and azithromycin regimens was commonly administered for new-onset BOS patients. Concomitant use of other immunosuppressive agents was administered for those who had extrapulmonary cGVHD. Once initial therapy failed, second-line treatment was administered, including tyrosine kinase inhibitors, ruxolitinib, and mesenchymal stem cells (MSCs).

### Definitions

Acute graft-versus-host disease (aGVHD) and cGVHD were graded according to the literature (25, 26). Relapse was defined by morphologic evidence in the peripheral blood, marrow, or extramedullary sites. Non-relapse mortality (NRM) was estimated as death without evidence of leukemia recurrence. OS was defined as the time from the first day of transplantation to death as a result of any cause.

### Statistical analysis

Baseline variables of the patients were described using number and percentage for categorical variables or median and range for continuous variables. Grouped variables were compared using the χ^2^ test, and continuous variables were compared using the non-parametric Mann–Whitney U test. OS was estimated using the Kaplan–Meier method. The log-rank test was used for group comparisons of survival distributions. The cumulative incidence rate of BOS, NRM, and relapse was estimated in the competing risk framework. Death was considered as a competing event for BOS, whereas relapse and NRM were treated as events competing with each other. All of the endpoints were measured from the date of transplantation. Groups were then compared using the Gray test^[21]^. The Cox proportional hazards model was used to explore risk factors of BOS in univariable and multivariate analyses. In this study, p < 0.05 for a 2-sided test was considered to be significant. All analyses were conducted using the statistical package R (http://www.r-project.org) and EmpowerStats software (www.empowerstats.com, X&Y Solutions, Inc., Boston, MA, USA).

## Results

### Patients’ clinical and transplant characteristics

A total of 1,405 patients with hematologic malignancies were enrolled in this retrospective study, including 551 patients with HID, 654 with MSD, and 200 with MUD transplants. There were 856 men and 549 women, with a median age of 31.0 [interquartile range (IQR), 22.0–42.0] years. There were 789 patients and 616 patients in the ATG and non-ATG groups, respectively. ATG prophylaxis was usually not applied to patients of the MSD group unless they participated in clinical trials. One hundred (15.3%) patients received ATG in the MSD group because of clinical trials. Primary diseases included 505 patients with lymphocytic malignancies and 830 patients with myelogenous malignancies. Five hundred fifty-one (92.7%) patients in the HID group and 65 (9.9%) patients in the MSD group received mixed grafts. All patients in the MUD group received PBSC grafts. The patients’ clinical and transplant characteristics are shown in **Table 1**.

**Table 1:** Patients’ clinical and transplant characteristics.

Variables	HID (n=551)	MSD (n=654)	MUD (n=200)	Total (n=1,405)	p–value
Patients’ age, median (IQR), years	30.0 (21.0–43.0)	32.5 (24.0–42.0)	28.0 (20.8–38.0)	31.0 (22.0–42.0)	0.001
Donors’ age, median (IQR),years	32.0 (22.0–45.0)	32.0 (24.0–42.0)	28.0 (24.0–36.0)	31.0 (23.0–42.0)	0.006
Patients’ gender, N (%)					0.244
Male	343 (62.3)	384 (58.7)	129 (64.5)	856 (60.9)	
Female	208 (37.7)	270 (41.3)	71 (35.5)	549(39.1)	
Donors’ gender, N (%)					< 0.001
Male	371 (70.9)	350 (54.2)	139 (78.1)	860 (63.8)	
Female	152 (29.1)	296 (45.8)	39 (21.9)	545(36.2)	
Primary diseases, N (%)					0.003
Lymphocytic malignancy	192 (34.8)	244 (37.3)	97 (48.5)	533 (37.9)	
Myelogenous malignancy	359 (65.2)	410 (62.7)	103 (51.5)	872 (62.1)	
Status at transplantation, N (%)					0.221
CR	434 (78.8)	490 (74.9)	158 (79.0)	1,082 (77.0)	
NR	117 (21.2)	164 (25.1)	42 (21.0)	323 (23.0)	
GVHD prophylaxis, ATG, N (%)					< 0.001
Yes	551 (100.0)	100 (15.3)	200 (100.0)	851 (60.6)	
No	0 (0.0)	554 (84.7)	0 (0.0)	554 (39.4)	
Conditioning regimen, N (%)					< 0.001
BU–based	292 (53.0)	377 (57.6)	72 (36.0)	741 (52.7)	
TBI–based	259 (47.0)	277 (42.4)	128 (64.0)	664 (47.3)	
Source of stem cell, N (%)					< 0.001
PBSC	40 (7.3)	589 (90.1)	200 (100.0)	821 (59.2)	
PBSC + BM	511 (92.7)	65 (9.9)	0 (0.0)	576 (40.8)	
CMVemia, N (%)					< 0.001
Yes	396 (71.9)	281 (43.0)	125 (62.5)	802(57.1)	
No	155 (28.1)	373 (57.0)	75 (37.5)	603 (42.9)	
aGVHD, N (%)					< 0.001
Yes	213 (38.7)	163 (24.9)	69 (34.5)	445 (31.7)	
No	338 (61.3)	491 (75.1)	131 (65.5)	960 (68.3)	
cGVHD, N (%)					0.522
Yes	201 (36.5)	225 (34.4)	77 (38.5)	503 (35.8)	
No	350 (63.5)	429 (65.6)	123 (61.5)	902 (64.2)	

CR, complete remission; NR, non–complete remission; PBSC, peripheral blood stem cell; BM, bone marrow; CMVemia, CMV DNA viremia. BU, busulfan; TBI, total body irradiation. IQR, interquartile range; aGVHD, acute graft-versus-host disease; cGVHD, chronic graft-versus-host disease; HID, haploidentical donor; MSD, HLA-matched sibling donor; MUD, matched unrelated donor.

### Bronchiolitis obliterans syndrome and chronic graft-versus-host disease

**Figure 1:**
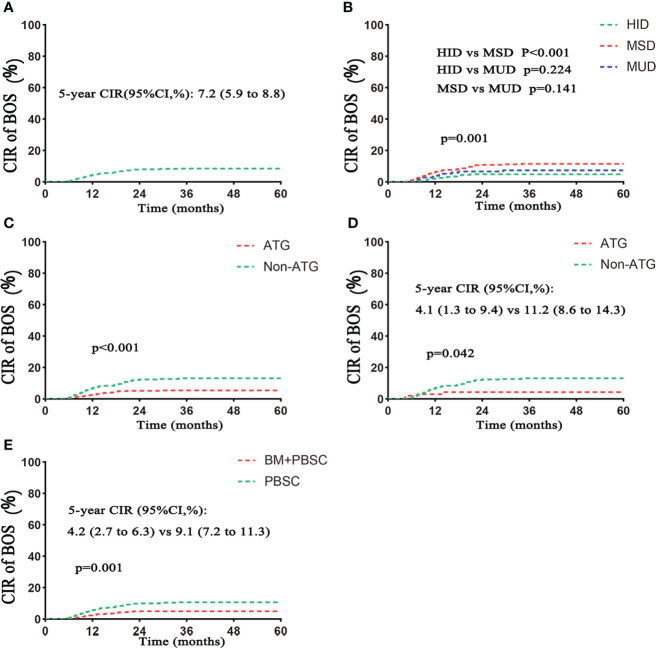
Cumulative incidence of BOS. **(A)** CIR of BOS in the total population. **(B)** CIR of BOS based on donor type. **(C)** CIR of BOS based on ATG. **(D)** CIR of BOS based on ATG in the MSD subgroup. **(E)** CIR of BOS based on grafts. CIR, cumulative incidence rate. Follow–up started from transplantation. HID, haploidentical donor; MSD, HLA-matched siblingdonor; MUD, matched unrelated donor; PBSC, peripheral blood stem cell; BM, bone marrow.

Eighty-eight patients developed BOS, including 18 patients in the HID group, 58 in the MSD group, and 12 in the MUD group. The cumulative incidences of BOS in the whole population were 3.9%, 6.9%, and 7.2% for 1 year, 2 years, and 5 years, respectively (**Figure 1A** **;** **Table 2**). The 5-year cumulative incidence of BOS was significantly lower in the HID group compared with that in the MSD group [4.1% (95% CI, 2.5%–6.2%) vs. 10.0% (95% CI, 7.7%–12.6%), p < 0.001; **Table 2**, **Figure 1B**]. The incidence of BOS was similar between HID and MUD transplantation (p = 0.224; **Figure 1B**). The 5-year cumulative incidence of BOS was lower in the ATG group than that in the non-ATG group in both the whole and MSD populations (4.6% vs. 11.2%, p < 0.001, and 4.1% vs. 11.2%, p = 0.042, respectively; **Table 2**; **Figures 1C, D**). The 5-year incidence of BOS in the mixed graft group was lower than that in the PBSC group (4.2% vs. 9.1%, p = 0.001).

**Table 2:** Outcomes.

Outcomes	Donor type (%, 95% CI)				GVHD prophylaxis (%, 95% CI)			Total
	HID	MSD	MUD	p–value	Non–ATG	ATG	p–value	
CIR of BOS								
1–year	2.1 (1.1–3.6)	5.5 (4.2–7.9)	3.6 (1.6–6.8)	0.004	6.4 (4.5–8.7)	2.5 (1.6–3.8)	0.002	3.9 (3.0–5.1)
2–year	4.1 (2.5–6.2)	9.5 (7.3–12.1)	5.6 (3.0–9.5)	<0.001	10.7 (8.1–13.6)	4.4 (3.1–6.1)	<0.001	6.9 (5.6–8.4)
5–year	4.1 (2.5–6.2)	10.0 (7.7–12.6)	6.2 (3.4–10.2)	<0.001	11.2 (8.6–14.3)	4.6 (3.2–6.3)	<0.001	7.2 (5.9–8.8)
CIR of cGVHD, 5–year	40.1 (36.2–45.3)	38.0 (34.1–42.0)	39.5 (32.6–46.4)	0.413	38.9 (34.6–42.3)	39.7 (36.1–43.2)	0.365	39.4 (36.6–42.1)
OS, 5–year	70.3 (66.0–74.8)	72.3 (68.7–76.0)	69.8 (63.6–76.5)	0.584	69.6 (65.6–73.9)	72.4 (69.2–75.7)	0.480	71.3 (68.8–73.9)

CIR, cumulative incidence rate, OS, overall survival. cGVHD, chronic graft-versus-host disease; HID, haploidentical donor; MSD, HLA-matched siblingdonor; MUD, matched unrelated donor.

**Figure 2:**
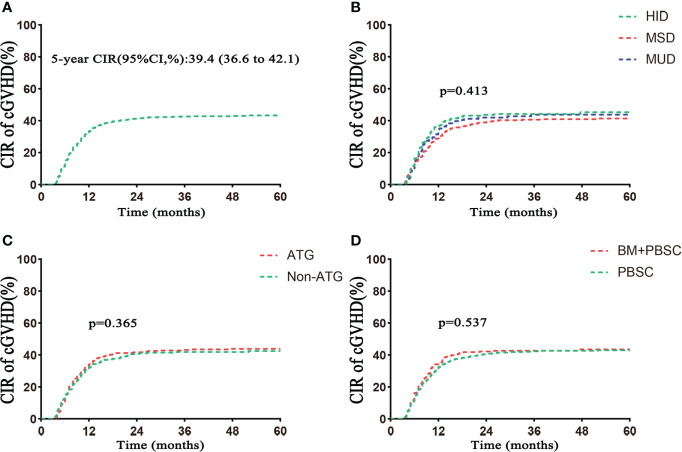
Cumulative incidence of cGVHD. **(A)** Cumulative incidence of cGVHD in the total population. **(B)** Cumulative incidence of cGVHD based on donor type. **(C)** Cumulative incidence of cGVHD based on ATG. **(D)** Cumulative incidence of cGVHD based on grafts. CIR, cumulative incidence rate. Follow–up started from transplantation.

Five hundred three patients developed cGVHD, including 201 in the HID group, 225 in the MSD group, and 77 in the MUD group (p = 0.522; **Table 1**). The 5-year cumulative incidence of cGVHD in the whole population was 39.4% (**Table 2**). There were similar 5-year cumulative incidences of cGVHD among HID, MSD, and MUD transplantation, respectively, 40.1%, 38.0%, and 39.5% (p = 0.413; **Table 2**, **Figure 2B**). The incidences of cGVHD between the ATG and non-ATG groups were not significantly different (39.7% vs. 38.9%, p = 0.365; **Figure 2C**). The grafts had no effect on the incidences of cGVHD (p = 0.537; **Figure 2D**).

### Risk factors for bronchiolitis obliterans syndrome

Univariate and multivariate analyses of BOS are shown in **Table 3**. Multivariate analysis showed that HID and ATG were the protective factors for BOS [odds ratio (OR) 0.3, 95% CI 0.2–0.6, p < 0.001 vs. MSD; OR 0.3, 95% CI 0.2–0.7, p = 0.001; **Table 3**). MUD transplantation presented a trend toward a lower risk of BOS compared with MSD transplantation (OR 0.5, 95% CI 0.2–1.1, p = 0.076; **Table 3**). MUD transplantation had a similar risk of BOS with HID transplantation (OR 1.7, 95% CI, 0.7–4.1, p = 0.205; **Table 3**). Mixed grafts indicated a lower risk than PBSC for BOS (0.3 OR, 95% CI 0.1–0.8, p = 0.013; **Table 3**). In addition, cGVHD and aGVHD of grades II–VI were independent risk factors for BOS (OR 10.1, 95% CI 4.0–25.0, p < 0.001; OR 2.1, 95% CI 1.1–4.3, p = 0.035; **Table 3**).

**Table 3:** Risk factors of BOS.

Variables	Univariate	p–value	Multivariate	p–value
Patients’ gender, Female vs. male	0.9 (0.6, 1.4)	0.644	1.0 (0.5, 2.0)	0.945
Donors’ gender, Female vs. male	1.1 (0.7, 1.7)	0.645	0.6 (0.3, 1.3)	0.222
Patients’ age, >31 years vs. <31 years^*^	1.0 (0.7, 1.6)	0.857	1.0 (0.4, 2.1)	0.927
Donors’ age, >31 years vs. <31 years^*^	1.0 (0.6, 1.5)	0.918	1.1 (0.5, 2.3)	0.792
Primary disease, Myelogenous vs. Lymphocytic	0.8 (0.5, 1.2)	0.290	0.8 (0.3, 1.9)	0.596
Status before transplantation, NR vs. CR	0.6 (0.3, 1.1)	0.101	0.7 (0.2, 2.6)	0.627
Conditioning, Non–BU vs. BU	1.0 (0.7, 1.6)	0.887	0.9 (0.4, 2.0)	0.771
Donor type				
HID vs. MSD	0.4 (0.2, 0.7)	**<0.001**	0.3 (0.2, 0.6)	**<0.001**
MUD vs. HID	1.6 (0.8, 3.2)	0.230	1.7 (0.7, 4.1)	0.205
MUD vs. MSD	0.6 (0.3, 1.2)	0.144	0.5 (0.2, 1.1)	0.076
Graft source, BM+PBSC vs. PBSC	0.5 (0.3, 0.8)	**0.003**	0.3 (0.1, 0.8)	**0.013**
GVHD prophylaxis, ATG vs. Non–ATG	0.4 (0.3, 0.7)	**<0.001**	0.3 (0.2, 0.7)	**0.001**
aGVHD, grades II–IV vs. 0–I	1.7 (1.0, 3.0)	**0.046**	2.1 (1.1, 4.3)	**0.035**
cGVHD, yes vs. no	12.2 (7.4, 20.1)	**<0.001**	10.1 (4.0, 25.0)	**<0.001**
CMVemia, yes vs. no	1.3 (0.8, 2.0)	0.259	1.5 (0.9, 2.4)	0.088

^*^Cutoff value was median number.

CR, complete remission; NR, not remission; BU, busulfan; HID, haploidentical donor; MSD, HLA–matched sibling donor; MUD, HLA–matched unrelated donor; BM, bone marrow; PBSC, peripheral blood stem cell; CMVemia, CMV DNA viremia; aGVHD, acute graft-versus-host disease; cGVHD, chronic graft-versus-host disease.

### Survival

**Figure 3:**
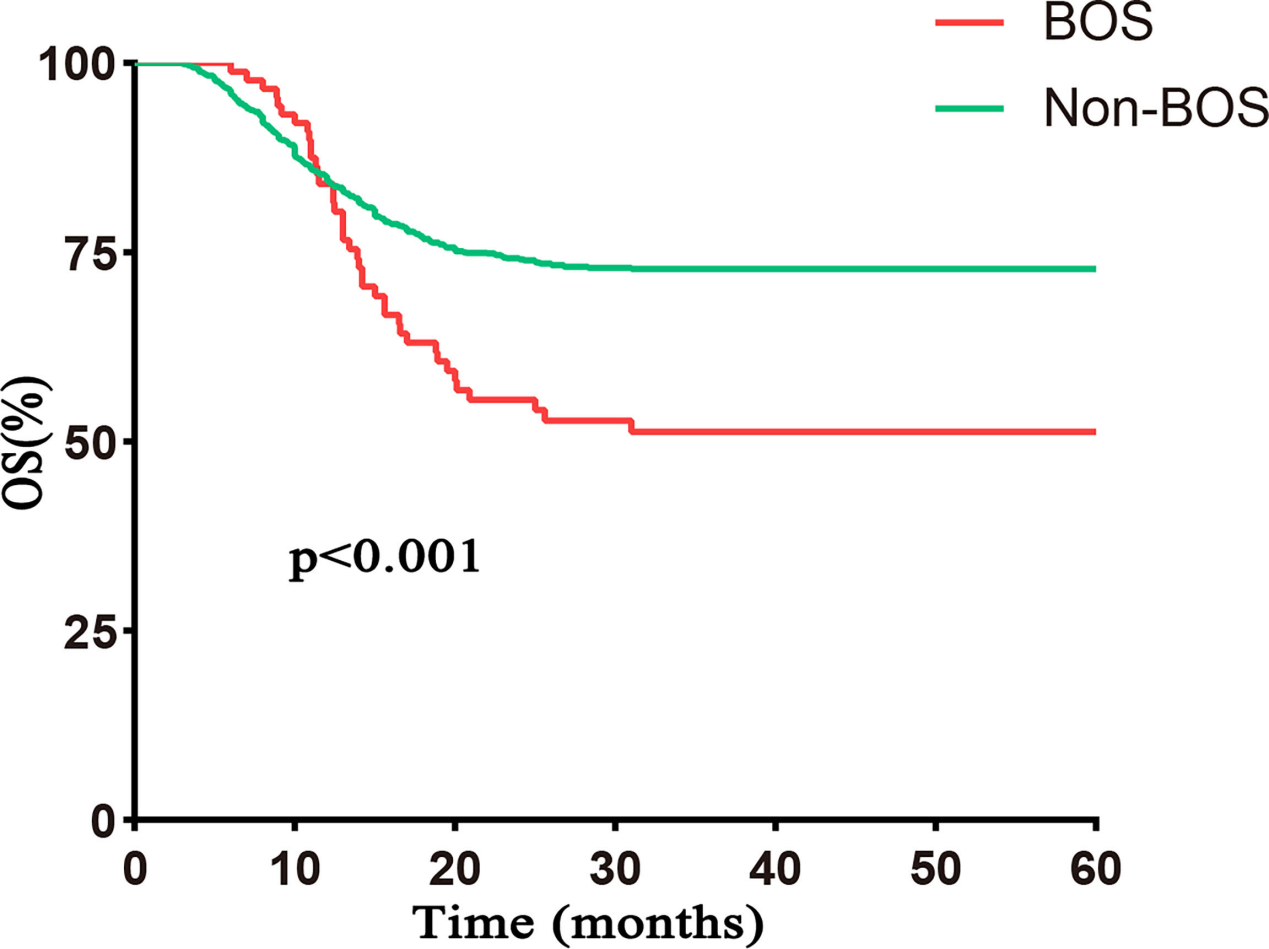
OS based on BOS. The 5–year OS was significantly worse in the BOS group than that in the non–BOS group [51.3% (95% CI 41.4%–63.5%) vs. 72.8% (95% CI 70.3%–75.5%), respectively, p < 0.001]. Follow–up started from transplantation.

With a median follow-up of 56.7 months (range, 3.5–60 months), 40 patients with BOS died. The causes of death included respiratory infections (n = 13), respiratory failure associated with BOS progression (n = 17), and non-pulmonary causes (n = 10). The 5-year OS posttransplantation was significantly different between BOS and non-BOS groups [51.3% (95% CI 41.4%–63.5%) vs. 72.8% (95% CI 70.3%–75.5%), respectively, p < 0.001; **Figure 3**].

**Figure 4:**
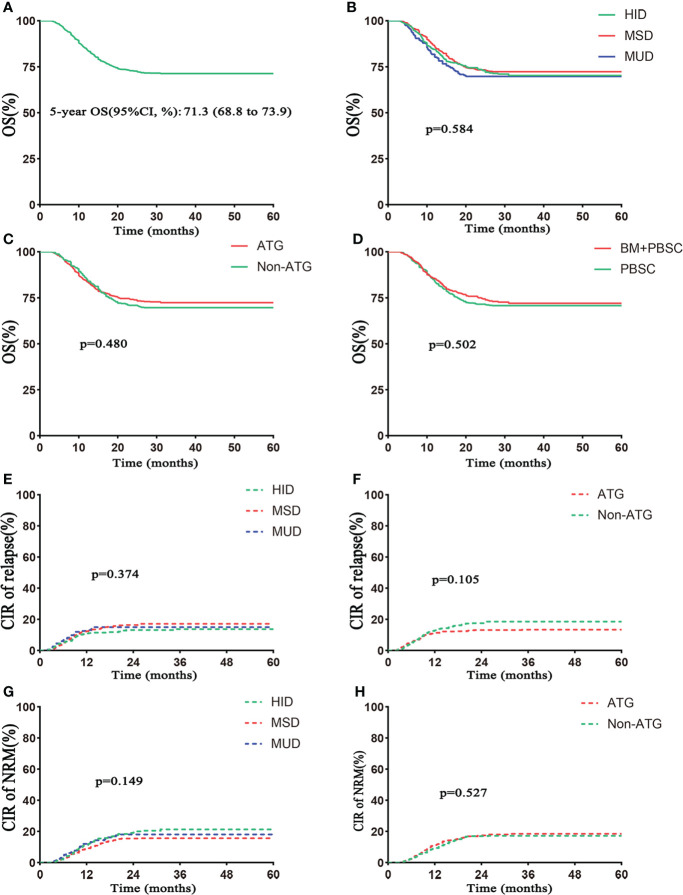
Outcomes of survival. **(A)** OS in the total number of patients. **(B)** OS based on donor type. **(C)** OS based on ATG. **(D)** OS based on grafts. The 5–year OS rates were 72.0% (95% CI 68.0%–76.3%) in the BM+PBSC group and 70.8% (95% CI 67.6%–74.1%) in the PBSC group. **(E)** Cumulative incidence of relapse based on donor type. **(F)** Cumulative incidence of relapse based on ATG. **(G)** Non–relapse mortality (NRM) based on donor type. **(H)** NRM based on ATG. Follow–up started from transplantation.

OS at 5-year posttransplantation was 71.3% (95% CI 68.8%–73.9%) in the whole population (**Table 2**, **Figure 4A**), and 5-year OS was 70.3% (95% CI 66.0%–74.8%) in the HID group, 72.3% (95% CI 68.7%–76.0%) in the MSD group, and 69.8% (95% CI, 63.6%–76.5%) in the MUD group (p = 0.584; **Table 2**, **Figure 4B**). The 5-year OS was similar between ATG and non-ATG groups [72.4% (95% CI 69.2%–75.7%) vs. 69.6% (95% CI 65.6%–73.9%), p = 0.480; **Table 2**, **Figure 4C**]. The effect of grafts on 5-year OS was not significant (mixed grafts vs. PBSC: 72.0% vs. 70.8%, p = 0.502; **Figure 4D**). Among the HID, MSD, and MUD groups, 5-year cumulative relapse rates were 12.7%, 15.9%, and 14.1% (p = 0.374; **Figure 4E**), and 5-year NRM rates were 19.9%, 14.8%, and 16.8%, respectively (p = 0.149; **Figure 4G**). Between the ATG and non-ATG groups, the 5-year cumulative relapse rates were not significantly different (p = 0.105), so was the 5-year NRM (p = 0.527).

## Discussion

It is currently unclear whether the incidence of BOS was comparable between HID and MSD transplantation. We retrospectively compared the cumulative incidence of BOS between HID transplantation based on ATG for GVHD prophylaxis and MSD transplantation. Our results showed that HID transplantation based on ATG had a lower incidence of BOS compared with MSD transplantation, which might be attributed to applications of ATG and mixed grafts.

A growing number of studies show that ATG can reduce the incidence of aGVHD and cGVHD, especially cGVHD, regardless of HID, MUD, or MSD transplantation (14, 15, 27, 28). It is well known that BOS is the manifestation of cGVHD in the lung (20). In the setting of MSD and MUD transplantation, some studies suggested that ATG was associated with a lower incidence of BOS (29–32). Regarding HID transplantation, our results showed that ATG–based HID transplantation also had a lower incidence of BOS than MSD transplantation, and ATG was a protective factor against BOS. Meanwhile, the incidence of BOS was also lower in MUD transplantation based on ATG prophylaxis than that in MDS transplantation. More importantly, in the subgroup of MSD transplantation, a small number of patients receiving ATG also had a lower incidence of BOS than patients not receiving ATG. Mechanistically, Hoegh–Petersen et al. (33) reported that ATG reduced the risk of aGVHD and cGVHD by inhibiting naive CD4 T cells and upregulating Treg cells. This might be one of the reasons for reducing the incidence of BOS. In addition, ATG induces the modulation of key functional molecules that mediate leukocyte and endothelium interactions and interferes with leukocyte adhesion to the endothelium, which might attenuate the degree of lung tissue injury and contribute to avoiding the occurrence of BOS (34). To sum up, we suggested that ATG might reduce the incidence of BOS, regardless of donor type.

The development of BOS was associated with many factors, such as patients’ age, ATG used, donor type, BU–based conditioning, GVHD, and CMVemia (29, 30, 35–37). In this study, we observed that HID transplantation, ATG prophylaxis, and mixed grafts were protective factors, whereas aGVHD and cGVHD were risk factors for BOS. Based on these findings, we speculated that the protective effect of HID transplantation against BOS was associated with applications of ATG and mixed grafts instead of itself. For the impact of ATG on BOS, studies by Gazourian et al. (29) and Duque–Afonso et al. (35) reported that ATG was a protective factor against BOS in patients receiving MSD–SCT and MUD–SCT. A case–control study (30) from China observed that ATG was a protective factor against BOS in HID recipients. Our result from a large sample size was consistent with these reported. For impact factors previously reported, such as GVHD, BU–based conditioning, and CMVemia, we found that they had no impact on the development of BOS except GVHD. The reasonable explanations are as follows. Our patients received intravenous BU, which would ensure complete bioavailability and reliable systemic drug exposure, with lower risks for severe pulmonary injury (38). In addition, our patients with CMVemia received preemptive antiviral therapy, which reduced risks of transformation of CMV pneumonia and pulmonary injury (39). A new finding in this study was that patients receiving mixed grafts had a lower risk of BOS than those who received PBSC grafts. This might be associated with lower incidences of GVHD in patients receiving mixed grafts (40, 41).

BOS presents early asymptomatic and insidious characteristics, leading to missing the timing of treatment. Even worse, the effective therapy for BOS remains lacking (20, 42). Thus, the mortality of BOS is high. In our study, patients with BOS had a significantly worse 5–year OS than that of patients without BOS, with 51.3% and 72.8%, respectively. The 5–year OS of patients with BOS was consistent with our previous report (43). Our results were superior to literature reported, in which a review of the literature showed that survival was 20% at 5 years (44). Improvement of survival might be associated with early application of MSCs for patients with BOS in our study. Our prospective study had demonstrated that MSCs were a safe and effective treatment for BOS patients posttransplantation to improve survival (43). In addition, whether application of ATG for GVHD prophylaxis increases relapse of patients posttransplantation remains a topic of intense discussion. A growing body of research has shown that an appropriate dose (4.5–10.0 mg/kg) of ATG will not increase the relapse rate of patients with hematologic malignancies (12, 14, 45). In this study, our results indicated that ATG for GVHD prophylaxis did not increase relapse of patients with hematologic malignancies as well.

In conclusion, we suggested that HID transplantation based on ATG for GVHD prophylaxis presented a lower incidence of BOS than MSD transplantation. This might be associated with applications of ATG and mixed grafts. This study might provide a potential strategy for preventing BOS posttransplantation regardless of HID, MUD, or MSD transplantation.

## Data availability statement

The original contributions presented in the study are included in the article/Supplementary Material. Further inquiries can be directed to the corresponding authors.

## Ethics statement

The studies involving human participants were reviewed and approved by The Ethics Committee of Nanfang Hospital. The patients/participants provided their written informed consent to participate in this study.

## Author contributions

QL and LX conceived the idea, GW, ZF, HX, and FH collected the data and gave critical suggestions, NX, HJ, SY, ZY, and JF gave valuable suggestions, GW performed statistical analysis and wrote the original draft. QL and LX revised the manuscript. All authors read and approved the final manuscript.

## Funding

This work was supported by the National Natural Science Foundation of China (Grant Nos. 81970161;82070190;8217011203), the Key Research and Development Projects of Guangdong Province (Grant No. 2019B020236004), the National Key Research and Development Projects (Grant No. 2021YFC2500302), Outstanding Youths Development Scheme of Nanfang Hospital, Southern Medical University (Grant No. 2021J010).

## Acknowledgments

We sincerely thank all colleagues in the Department of Hematology, Nanfang Hospital, for their help in this study.

## Conflict of interest

The authors declare that the research was conducted in the absence of any commercial or financial relationships that could be construed as a potential conflict of interest.

## Publisher’s note

All claims expressed in this article are solely those of the authors and do not necessarily represent those of their affiliated organizations, or those of the publisher, the editors and the reviewers. Any product that may be evaluated in this article, or claim that may be made by its manufacturer, is not guaranteed or endorsed by the publisher.
